# Limited genetic diversity in the PvK12 Kelch protein in *Plasmodium vivax* isolates from Southeast Asia

**DOI:** 10.1186/s12936-016-1583-0

**Published:** 2016-11-08

**Authors:** Meilian Wang, Faiza Amber Siddiqui, Qi Fan, Enjie Luo, Yaming Cao, Liwang Cui

**Affiliations:** 1 Department of Microbiology and Parasitology, College of Basic Medical Sciences, China Medical University, 77 Puhe Road, Shenbei New District, Shenyang, 110013 China; 2 Department of Entomology, Pennsylvania State University, 501 ASI Building, University Park, PA 16802 USA; 3 Dalian Institute of Biotechnology, Dalian, Liaoning Province China; 4 Department of Immunology, College of Basic Medical Sciences, China Medical University, 77 Puhe Road, Shenbei New District, Shenyang, 110013 China

**Keywords:** Artemisinin resistance, Kelch domain protein, *Plasmodium vivax*, Malaria, Genetic diversity, PfK13, PvK12

## Abstract

**Background:**

Artemisinin resistance in *Plasmodium falciparum* has emerged as a major threat for malaria control and elimination worldwide. Mutations in the Kelch propeller domain of PfK13 are the only known molecular markers for artemisinin resistance in this parasite. Over 100 non-synonymous mutations have been identified in PfK13 from various malaria endemic regions. This study aimed to investigate the genetic diversity of PvK12, the *Plasmodium vivax* ortholog of PfK13, in parasite populations from Southeast Asia, where artemisinin resistance in *P. falciparum* has emerged.

**Methods:**

The PvK12 sequences in 120 *P. vivax* isolates collected from Thailand (22), Myanmar (32) and China (66) between 2004 and 2008 were obtained and 353 PvK12 sequences from worldwide populations were retrieved for further analysis.

**Results:**

These PvK12 sequences revealed a very low level of genetic diversity (π = 0.00003) with only three single nucleotide polymorphisms (SNPs). Of these three SNPs, only G581R is nonsynonymous. The synonymous mutation S88S is present in 3% (1/32) of the Myanmar samples, while G704G and G581R are present in 1.5% (1/66) and 3% (2/66) of the samples from China, respectively. None of the mutations observed in the *P. vivax* samples were associated with artemisinin resistance in *P. falciparum*. Furthermore, analysis of 473 PvK12 sequences from twelve worldwide *P. vivax* populations confirmed the very limited polymorphism in this gene and detected only five distinct haplotypes.

**Conclusions:**

The PvK12 sequences from global *P. vivax* populations displayed very limited genetic diversity indicating low levels of baseline polymorphisms of PvK12 in these areas.

**Electronic supplementary material:**

The online version of this article (doi:10.1186/s12936-016-1583-0) contains supplementary material, which is available to authorized users.

## Background

Malaria is a major public health problem in the Greater Mekong Subregion (GMS), including Cambodia, China, Laos, Myanmar, Thailand, and Vietnam [1]. Malaria in these GMS countries is concentrated along the international borders. Since 2001, artemisinin (ART) combination therapy (ACT) has been recommended as the first-line treatment in the national treatment guidelines of most malaria endemic countries and have played an important role in reducing global malaria-associated mortality and morbidity. However, the recent emergence of ART resistance in *Plasmodium falciparum* in the GMS is extremely concerning. *P. falciparum* isolates resistant to ARTs were first detected in this region in 2008 [2]. Since then, ART resistance has spread and/or emerged in other areas of the GMS [3–6]. ART resistance is defined as the parasite clearance half-life of >5 h or presence of parasites in patients 3 days after ART treatment. Recently, mutations in the propeller domain of the *Kelch 13* (K13) gene from *P. falciparum* (PF3D7_1343700) were shown to be associated with in vitro resistance to ART as well as in vivo delayed parasite clearance [7]. Kelch-like proteins consist of a series of four to seven Kelch motifs which interact with different binding partners, thereby mediating a wide variety of cellular functions [8]. Some Kelch proteins also act as substrate adaptors for the cullin 3 ubiquitin ligases [9], but their exact functions in *Plasmodium* remain to be elucidated. In a transcriptomics study, Mok et al. have shown that the parasites with mutated K13 have an upregulated unfolded protein response pathway [10]. Another study has shown that ART acts via the parasite’s cell stress response involving the ubiquitin/proteasome system, which is enhanced by certain *k13* mutations [11]. Furthermore, phosphatidylinositol-3-kinase (PI3K)-mediated signaling has been identified as a probable target of ART, and K13 has been shown to regulate the levels of PI3K in parasites [12]. More studies are required in order to delineate the underlying molecular mechanism of K13-mediated ART resistance. Following the identification of *k13* gene as a molecular marker for ART resistance [7, 13], numerous studies have been performed to assess the polymorphism in this gene from various malaria endemic regions [14–21]. Several mutations in the Kelch propeller domain have now been associated with in vitro ring-stage survival assays and delayed parasite clearance rates in patients treated with ARTs [7, 13, 22]. Consequently, sequencing the Kelch propeller domain of the *k13* gene has become an important tool in the surveillance of ART resistance in *P. falciparum*. A total of 186 *k13* alleles, including 108 nonsynonymous mutations, have been reported so far in *P. falciparum* [23]. There is significant geographic heterogeneity in both the patterns of the *k13* mutations and their prevalence across the GMS [23], possibly reflecting different drug histories and evolutionary origins. Fortunately, the resistance associated mutations are still confined to Southeast Asia. Some rare alleles are found in other regions but are not associated with ART resistance [23–26].

While *P. falciparum* is responsible for the majority of malaria-related mortality, *Plasmodium vivax* is the most prevalent parasite species outside of Africa. *Plasmodium vivax* caused an estimated 13.8 million cases globally in 2015, and accounted for about half of all malaria cases outside Africa [27]. Although chloroquine (CQ) remains the primary treatment option for *P. vivax*, ACT is used in places such as Indonesia where CQ resistance is evident in this parasite [28]. To date, there are no reports of clinical resistance of *P. vivax* to ARTs. Yet, in areas with co-endemicity of *P. falciparum* and *P. vivax*, mixed infections often occur at high prevalence [29]. ACT is also used to treat mixed-species infections [30, 31]. As a result, *P. vivax* may have been under similar drug selective pressure as *P. falciparum*. For example, point mutations in *pvdhfr* and copy number variations in *pvmdr1* may reflect past drug histories of pyrimethamine and mefloquine, respectively, which have been used to treat falciparum malaria [32–34]. Therefore, it would be interesting to determine whether ART drugs have imposed similar selection on *PvK12* gene. PvK12 is the ortholog of the PfK13, and is present on chromosome 12 of *P. vivax* [35]. A recent study showed that a nonsynonymous mutation in the PvK12 gene circulates at very low frequencies in Cambodia where ART resistance in *P. falciparum* first emerged [35]. Thus, this study aims to characterize the baseline genetic variability of PvK12 gene in parasite populations from various regions in the GMS collected in 2004–2008, before the first reports of ART resistance in *P. falciparum*.

## Methods

### Collection of *Plasmodium vivax* clinical samples

Clinical *P. vivax* samples were collected from patients with acute *P. vivax* malaria in central China (66), northeast Myanmar (32) and western Thailand (22) in 2004–2008. Diagnosis was conducted by microscopy and finger-prick blood samples from confirmed *P. vivax* patients were blotted onto Whatman filter papers. Since central China is only endemic for *P. vivax* malaria and treatment has always been CQ-primaquine, parasite population from this region would have never been exposed to ART drugs. These parasite samples would be controls for the baseline mutations in the PvK12 gene. In Thailand, artesunate has been used since 1990s mostly in combination with mefloquine [36, 37], whereas at the China–Myanmar border, ART family drugs began to be deployed in late 1970s mostly as monotherapies until 2005. Therefore, *P. vivax* parasites from Thailand and northeast Myanmar are expected to have been exposed to ART drugs since mixed-species infections were deemed relatively prevalent in these areas [29].

### Sequencing of *P. vivax* K12 gene

**Fig. 1:**
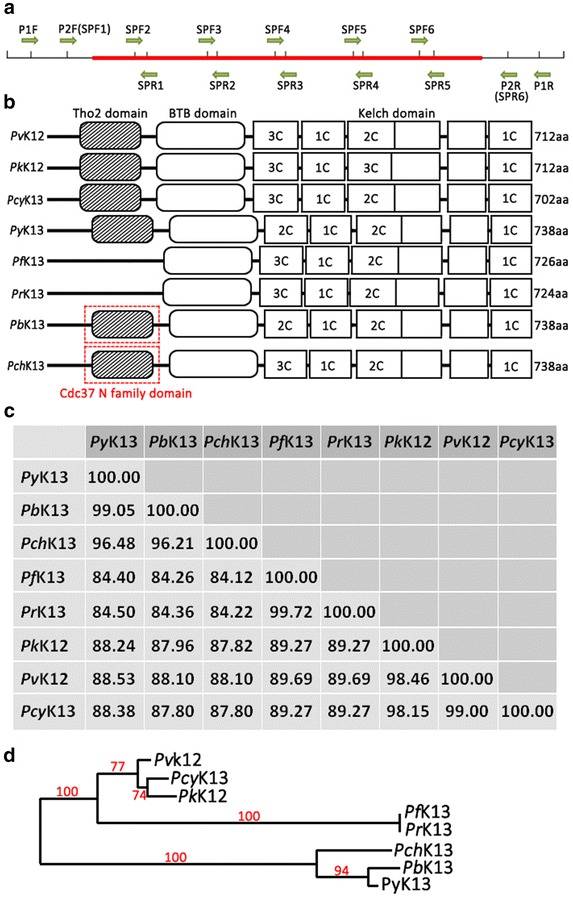
Domain organization and conservation of K13 protein across different *Plasmodium* species. **a** The PvK12 gene is shown in *red*. P1F and P1R shows the primer set used for the primary PCR. P2F and P2R shows the primers used for nested PCR for the amplification of full length PvK12. All the amplification primers used are present outside the ORF. SPF1, SPR1, SPF2, SPR2, SPF3, SPR3, SPF4 and SPR4 show the primers used for sequencing. **b** Schematic domain organization of Kelch protein in each species showing the BTB domain and six Kelch domains. The numbers of cysteine residues in each Kelch domain is indicated. Additional Tho2 and Cdc_37 N kinase binding domains are also shown. **c** Percentage of sequence similarity between amino acid sequences of seven *Plasmodium* species. **d** Neighbor-Joining tree of Kelch protein amino acid sequences from seven *Plasmodium* species. Bootstrap values generated from 1000 replicates are shown

Parasite genomic DNA was isolated from the dried blood spots using the QiaAmp DNA Mini Kit (Qiagen, Germany) according to the manufacturer’s instructions. The extracted DNA was eluted in 30 μl of TE buffer (10 mM Tris–HCl, 0.1 M EDTA, pH 8.0) and stored at −20 °C until use. A nested PCR approach was designed to amplify the full-length PvK12 gene (Fig. 1) using the primers shown in Additional file 1. Primary PCR amplification was performed with a 25 μl reaction mixture containing 1 μl of gDNA, 0.2 µM each primer, 2.5 mM MgCl_2_, and 0.3 µl Advantage^®^ 2 DNA polymerase (Clontech, Japan) under the following conditions: 94 °C for 2 min, followed by 35 cycles at 94 °C for 20 s, 62 °C for 30 s, and 65 °C for 2 min, and a final extension at 65 °C for 5 min. Nested PCR amplifications were performed in a 25 μl reaction mixture with 1 μl of the primary PCR product, 0.2 µM each primer, 2.5 mM MgCl_2_, and 0.3 μl Advantage^®^ 2 DNA polymerase under the following conditions: 94 °C for 2 min, followed by 35 cycles at 94 °C for 20 s, 60° C for 30 s, and 65 °C for 2 min, and a final extension at 65 °C for 5 min. The amplified PCR products were detected on a 1% agarose gel, and the sizes of the PCR products were estimated based on a 1 kb DNA ladder (NEB, USA). All PCR products were purified with the QIAquick Gel Extraction Kit (Qiagen, Germany) followed by sequencing in both directions using sequencing primers shown in Additional file 1. Sequences were assembled using Vector NTI (Invitrogen, USA) with manual editing.

### Phylogeny of PfK13 orthologs

Orthologs of PfK13 from other malaria parasite species *P. vivax* (PVX_083080); *Plasmodium reichenowi* (PRCDC_1342700); *Plasmodium chabaudi* (PCHAS_1361300); *Plasmodium berghei* (PBANKA_1356700); *Plasmodium yoelii* (PY17X_1362400); *Plasmodium knowlesi* (PKNH_1257700); and *Plasmodium cynomolgi* (PCYB_122000) were retrieved from PlasmoDB [38]. Sequences were aligned using CLUSTALW with manual editing. All the analyses in this study were done using MEGA6. A phylogenetic tree was constructed using the Neighbour-Joining method with 1000 pseudo-replications. Domain organization of PfK13 orthologs were analysed by using Batch CD-search tool in NCBI database, with E value cut off 0.10 [39].

### Kelch protein genetic diversity

To understand the global distribution of diversity in the *PvK12* gene, 353 published sequences were retrieved from PlasmoDB, including Cambodia (n = 284) [35], Thailand (n = 14), China (n = 5), North Korea (n = 1), Columbia (n = 22), Mexico (n = 10), Peru (n = 10), India (n = 2), Papua New Guinea (n = 3), Brazil (n = 1) and Mauritania (n = 1) (Additional file 2). All sequences were aligned against the Sal-1 reference sequence (PVX_083080) using MEGA6 [40]. Mutation in the PvK12 protein was mapped to the PvK12 protein 3-D structure predicted by using the I-TASSER online protein structure prediction tool [41] with the PfK13 protein structure as the template (ProteinDataBank:4YY8). Nucleotide diversity (π) and haplotype diversity (Hd) of PvK12 were determined by MEGA 6. The number of haplotypes was estimated from all the isolates and the Median-Joining method in NETWORK v4.6.1.3 program (fluxus-engineering.com) was used to establish genetic relationship among the PvK12 haplotypes [42].

### Natural selection and tests of neutrality

The rates of synonymous (dS) and nonsynonymous (dN) mutations were estimated using the modified Nei and Gojobori method [43] and significance was compared by the Z-test (P < 0.05). Tajima’s D [44] was determined using DnaSP 5.10.00, which tests the departure from the neutral theory of evolution with the assumption that the population size was constant. Significantly positive Tajima’s D values indicate a recent population bottleneck or balancing selection, whereas negative values suggest population expansion or directional selection. McDonald–Kreitman (MK) test, which compares the ratio of non-synonymous to synonymous substitutions (dN/dS) with polymorphic difference (within species; K_S_) and fixed difference (between closely related species; Ka) was used to examine the departure from neutrality using either *P. knowlesi* (PkK12) or *P. cynomolgi* (PcyK12) ortholog as the outgroup [45]. A sliding window approach was also used to identify specific regions of PvK12 that deviate from neutral expectations using a window size of 10 and a step size of 5 bp.

## Results

### Conservation of the Kelch proteins across different *Plasmodium* species

Kelch proteins are a widespread group of proteins that contain multiple Kelch motifs that form a β-propeller tertiary structure. These Kelch-β-propellers are generally involved in protein–protein interactions. In *Plasmodium*, the PfK13 orthologs also contain a BTB/POZ (Broad-Complex, Tramtrack, and Bric-a-Brac/Poxvirus and Zinc finger) domain in addition to the multiple Kelch motifs (Fig. 1b). Interestingly, there are two other conserved functional domains present in some of these *Plasmodium* orthologs (Fig. 1b; Additional file 3). Kelch proteins of *P. vivax*, *P. berghei*, *P. chabaudi*, *P. cynomolgi*, *P. knowlesi*, and *P. yoelii* have the Tho2-like (transcription factor/nuclear export subunit protein 2) domain. THO domain-containing proteins (in complex with other proteins) have function in messenger ribonucleoprotein metabolism and play a role in preventing transcription-associated genetic instability [46, 47]. Furthermore, in *P. berghei* and *P. chabaudi*, a Cdc37_N terminal kinase binding domain is also present (Fig. 1b). Cdc37 is a molecular chaperone required for the activity of numerous eukaryotic protein kinases, and this domain is present in the heat shock protein 70 (hsp 70) protein of *P. falciparum*. Alignment of Tho2 and Cdc37 N domains with respective conserved domain protein families is shown in Additional file 3. The full-length K13 protein sequences from the eight *Plasmodium* species vary from 712 to 738 aa (Fig. 1b). To estimate the evolutionary history of Kelch proteins among different *Plasmodium* species, the PfK13 orthologs from *P. vivax*, *P. berghei*, *P. chabaudi*, *P. cynomolgi*, *P. knowlesi*, *P. reichenowi* and *P. yoelii* were used for analysis. These proteins are evolutionarily conserved among the different *Plasmodium* species with high sequence similarity. Alignment of these eight Kelch proteins showed that both the BTB and Kelch domains are highly conserved (Additional file 4). The sequence similarity was the highest between *P. falciparum* and *P. reichenowi* (99.72%), and lowest between *P. falciparum* and *P. chabaudi* (84.68%) (Fig. 1c). Phylogenetic tree generated from eight Kelch protein sequences revealed three monophyletic branches, which conforms to earlier report of the phylogeny of the *Plasmodium* group based on other genetic markers [48]. The three rodent parasites grouped together, while *P. falciparum* was clustered with *P. reichenowi*. The other three species *P. vivax*, *P. cynomolgi* and *P. knowlesi* were branched together (Fig. 1d).

The *Plasmodium* Kelch protein shows homology with the human KEAP1 protein. KEAP1 is a substrate adaptor protein for an ubiquitin ligase complex that targets the Nrf2 transcription factor for degradation [49]. C151, C273, and C288 in the KEAP1 protein have been shown to be crucial for its interaction with Nrf2 in humans [50]. C151 lies in the BTB domain, while C273 and C288 lie in the linker region between BTB and Kelch domains of KEAP1 (Additional file 5). Alignment of Human KEAP1 with PvK12 shows that these cysteine residues are not conserved in the *Plasmodium* Kelch proteins (Additional file 5). In *Plasmodium*, the 6–8 cysteine residues are present only in the Kelch domains and are conserved across the different *Plasmodium* species (Fig. 1b; Additional file 4).

### Genetic diversity of PvK12 in *P. vivax* isolates from Southeast Asia

**Fig. 2:**
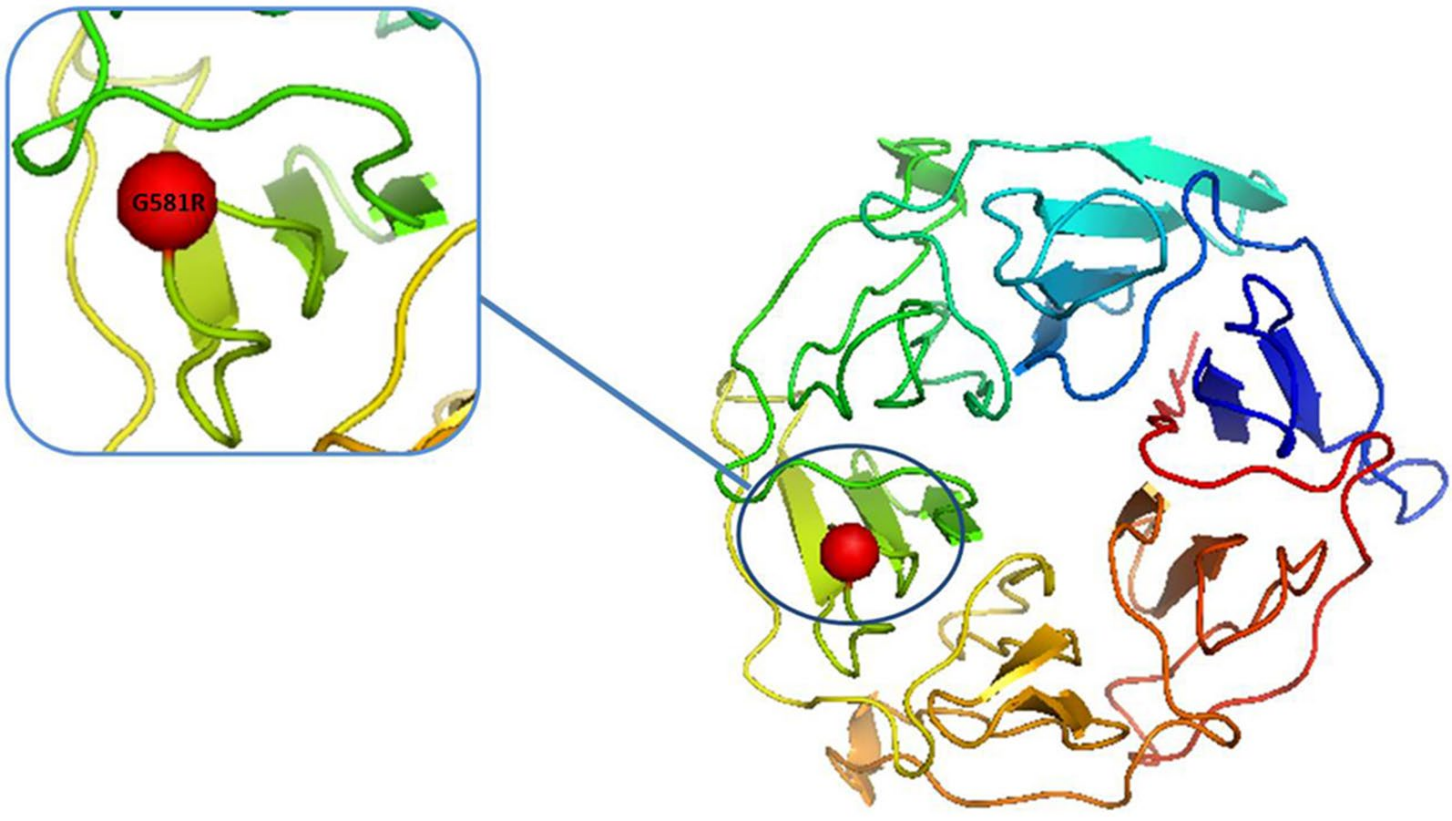
Structure modelling of *Plasmodium vivax* K12 propeller domain. 3D model of the six propeller blades was predicted by the I-TASSER software using the PfK13 protein structure as the template (Protein Data Bank: 4YY8). The non-synonymous mutation G581R is highlighted in red and lies in the third blade of the β-propeller domain

The full-length *PvK12* gene from 120 *P. vivax* isolates from Southeast Asia including Thailand (22), Myanmar (32) and central China (66) collected between 2004 and 2008 was amplified and sequenced. The full-length open reading frame (ORF) of PvK12 is 2139 bp encoding 712 amino acids (aa). Three mutations were observed in the samples when compared to the Sal-I reference sequence. Out of these mutations only one was nonsynonymous that resulted in an amino acid change (G581R). G581R and the synonymous mutation G704G were both found in the samples from China in 1.5% (1/66) and 3% (2/66) isolates respectively, whereas the other synonymous mutation S88S was present in one of the 32 samples from Myanmar. No mutations were identified in the Thai samples. The 120 parasite samples exhibited a very low level of genetic diversity (π < 0.00004) (Table 1). The G581R mutation lies in the propeller region of PvK12 protein (Fig. 2). The two synonymous mutations are present outside the Kelch domains. None of the mutations observed in these samples have been associated with ART resistance in *P. falciparum*.

**Table 1:** Single nucleotide polymorphisms and summary statistics of PvK12 in different geographical regions (*n* number of isolates, *s* number of SNPs, *H* haplotypes, *Hd* haplotype diversity, *Ns* number of non-synonymous, *Ss* number of synonymous

	n	s	H	π	θ	Hd	Ns	Ss	Tajima’s D	dN-dS
China	66	2	3	0.00004	0.00020	0.089	1	1	−1.31509	−0.9
Thailand	22	0	1	0.00000	0.00000	0.000	0	0	NA	0
Myanmar	32	1	2	0.00003	0.00012	0.063	0	1	−1.14244	−1.1
Cambodia	284	1	2	0.0000065	0.000075	0.014	1	0	−0.87436	1.1
Others	69	0	1	0.00000	0.00000	0.000	0	0	NA	0
Total	473	4	5	0.00001	0.00028	0.025	2	2	−1.52620	−1.0

### Worldwide population genetic diversity of PvK12

**Fig. 3:**
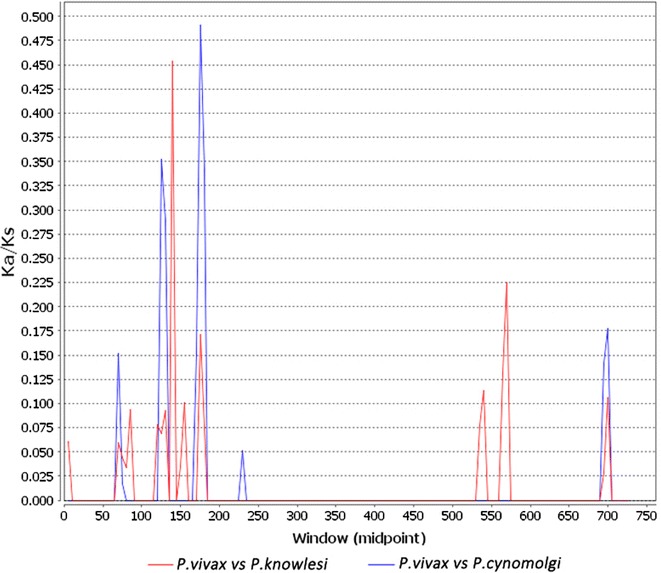
Within- and between-species divergence of PvK12. Sliding window analysis of Ka/Ks (non-synonymous divergence/synonymous divergence) calculated between *P. vivax* (PVX_083080) versus *P. knowlesi* (PKNH_1257700); *P. cynomolgi* (PCYB_122000). Window size of 10 bp and step size of 5 bp were used

Tajima’s D test performed on 473 PvK12 sequences obtained in the world did not detect potential natural selection on this gene (Table 1). In isolates from China, Myanmar and Cambodia, the Tajima’s D values were negative, suggesting the occurrence of rare alleles at low frequencies in these populations. Moreover, in these populations dN was significantly lower than dS, giving rise to negative ‘dN-dS’ values. MK test was used for comparing interspecific divergence (Ka/KS) using sequences from two phylogenetically related species *P. cynomolgi* and *P. knowlesi*. Ka/Ks ratio of >1 indicates positive selection, whereas Ka/Ks ratio between 0 and 1 shows neutral or disadvantageous mutations. A sliding window for Ka/Ks obtained by comparing the *P. vivax* sequences to sequences of *P. cynomolgi* and *P. knowlesi* gave Ka/Ks values of <0.5 in the full-length PvK12 protein indicating the absence of selection in this protein (Fig. 3). The Ka/Ks values were slightly higher in the N-terminal region before the BTB domain and the C-terminus after the Kelch domain, as compared to the rest of the protein. Further, the third Kelch motif has Ka/Ks values ranging between 0.1 and 0.2 when compared to *P. knowlesi*, whereas it is close to zero in the rest of protein sequence. This again confirms that the BTB and Kelch domains are highly conserved across different *Plasmodium* species.

To evaluate the population division of the worldwide *P. vivax* populations based on PvK12, the 120 sequences from this study were analysed along with 353 available PvK12 sequences from twelve global *P. vivax* populations, including sequences from five Asian (Thailand, China, Myanmar, Cambodia, India and North Korea), three American (Peru, Mexico, Columbia) and one African (Mauritania) countries. Genetic differentiation between worldwide populations was assessed by the haplotype network analysis (Additional file 6). Only five distinct haplotypes were observed including the wild type as the predominant. These haplotypes appear to have emerged independently in the Asian populations at very low frequencies as evidenced by the haplotype network (Additional file 6).

## Discussion

Anti-malarial drugs used to treat falciparum malaria have a significant impact on sympatric *Plasmodium* species, such as *P. vivax*, which is the second major cause of malaria in humans. As no reliable molecular markers associated with *P. vivax* drug resistance have been identified yet, it remains difficult to assess the antimalarial drug resistance of *P. vivax* from clinical studies. Therefore, it is important to identify drug resistance markers that can be used to determine the emergence and spread of resistance in *P. vivax*. The primary treatment for *P. vivax* consists of two drugs CQ and primaquine; but resistance to CQ was reported in many endemic areas [28]. In the sample collection areas of this study, CQ/primaquine remains as the primary treatment of vivax malaria with high efficacy. There were only sporadic reports of clinical CQ resistance in *P. vivax* in western Thailand [51] and the Thai–Myanmar border [52], though recent studies in northeast Myanmar suggested deteriorating CQ efficacy for treatment of *P. vivax* malaria [53].

The Kelch family proteins in *Plasmodium* consist of six conserved Kelch motifs and the BTB domain. The *Plasmodium* Kelch proteins are highly conserved across different species and are most similar to the human KEAP1 protein which is the major upstream regulator of a transcription factor (Nrf2) in humans [49]. KEAP1 binds the Cullin3 ubiquitin ligase via its N-terminal BTB domain and central linker domain, while the C-terminal Kelch domain of KEAP1 binds to Nrf2 [54]. Upon exposure to oxidative stress, ubiquitination of Nrf2 is inhibited that leads to the increased transcription of cytoprotective antioxidant responsive elements dependent genes [55]. Furthermore, C151, C273, and C288 in human KEAP1 have been shown to be important for Nrf2 ubiquitination [50]. To investigate whether malaria parasites employ a similar molecular mechanism, we aligned KEAP1 protein sequence from humans with PvK12 which revealed that these Cys residues are not conserved in *Plasmodium* (Additional file 5), while 6–8 conserved Cys residues are present in the Kelch domain in the malaria parasite. In addition the conserved domain analysis showed the presence of Tho2 domain and the Cdc37_N terminal kinase binding domain in some of the *Plasmodium* Kelch protein orthologs (Fig. 1b; Additional file 3). THO domain containing proteins function in mRNA transcription, processing and nuclear export of spliced mRNAs [56], whereas Cdc37_N terminal kinase binding domain acts as a molecular chaperone essential for the function of numerous eukaryotic protein kinases [57]. It would be interesting to unravel the cellular functions of the Kelch proteins with such domains in the malaria parasites.

Reports from the GMS have associated mutations in the propeller domain of PfK13 with ART resistance. According to a previous report by Popovici et al. PvK12 polymorphism in Cambodia was very limited compared to that of the *P. falciparum* K13 gene. Only 2 out of the 284 samples had the same nonsynonymous mutation at codon 552 (V552I) in samples from 2013 [35]. The current study tried to determine the genetic polymorphism in full-length PvK12 sequences from 120 *P. vivax* clinical isolates from China, Thailand and Myanmar collected in 2004–2008. The V552I mutation was not detected, while two synonymous (S88S, G704G) and one nonsynonymous (G581R) mutations were identified in these samples. All mutations were present at very low prevalence: S88S in 3% Myanmar samples, G704G in 1.5% China samples, and G581R in 3% China samples. Interestingly, though the G581R mutation drastically changes the property of the residue, it corresponds to the G595S mutation reported in PfK13 in *P. falciparum* samples from Mali [58], which has not been associated with ART resistance. Since there is no clinical outcome or resistance phenotype information for the *P. vivax* isolates, it is not clear whether the G581R mutation has any functional significance. It should be pointed out that the G581R mutation were present only in the central China samples, where ART drugs have never been used since this region is only endemic for vivax malaria. Therefore, without clinical resistance of *P. vivax* parasites to ART drugs, the point mutations observed in the world *P. vivax* populations could well represent background mutations. This finding is consistent with the low-level baseline mutations identified by Mita et al. in *P. falciparum* populations before the deployment of ACT [59].

In agreement with a previous report from Cambodia [35], the current results also showed limited baseline sequence diversity in PvK12 in additional GMS countries. Further analysis using a total of 473 PvK12 sequences from twelve worldwide *P. vivax* populations from 2001 to 2013 confirmed a very low level of genetic diversity as compared to that of the PfK13 gene. This result implies that PvK12 sequences from these areas are highly conserved with very low level of baseline polymorphisms. Since our samples used were collected in 2004–2008, it is necessary to conduct follow-up studies using parasite samples collected in more recent years in these regions such as Indonesia where intensive deployment of ACT for the treatment of vivax malaria has occurred.

## Conclusions

Analysis of these samples along with 473 PvK12 sequences from twelve worldwide *P. vivax* populations collected in 2001–2013, revealed a low baseline level of PvK12 polymorphisms.

## Additional files

**Additional file 1: Table S1.** Primers used for amplification and sequencing of the full-length PvK12 gene.

**Additional file 2: Table S2.** Accession numbers of *Plasmodium vivax* Kelch protein K12 (*Pv* K12) sequences retrieved from Plasmodb.

**Additional file 3.** Alignment of the Tho2 and Cdc37 N domains. Alignment of the Tho2 and Cdc37 N domains of *Plasmodium* with the THo2 and Cdc37 N conserved domain family proteins. The hyphens show the gaps in the alignment.

**Additional file 4.** Multiple sequence alignment of Kelch protein from seven different *Plasmodium* species. Clustal Omega server was used to align the Kelch proteins from *P. falciparum* (PF3D7_1343700); *P. reichenowi* (PRCDC_1342700); *P. chabaudi* (PCHAS_1361300); *P. berghei* (PBANKA_1356700); *P. yoelii* (PY17X_1362400); *P. knowlesi* (PKNH_1257700); *P. cynomolgi* (PCYB_122000), *P. vivax* (PVX_083080). The BTB and the Kelch domains are boxed in red and blue respectively. Cysteine residues are highlighted in green. The dots indicate the break in the sequences. The BTB domains starting from 327 are shown and boxed in red. The Kelch domains are shown in blue box. The hyphens show the gaps in the alignment.

**Additional file 5.** Alignment of PvK12 with human KEAP1. The BTB and Kelch domains are boxed for both the proteins. The Pvk12 BTB domain is boxed in blue and Kelch domain in green; the human KEAP1 BTB domain is boxed in red and Kelch domain in pink, respectively. All the cysteines are highlighted in red. C151, C273, C288 of KEAP1 are highlighted in yellow. The hyphens show the gaps in the alignment.

**Additional file 6.** Haplotype network for PvK12 from global *P. vivax* populations. The size of the pies reflects the frequency of a particular haplotype. The lengths of the lines connecting the pies, measured from their centres, are in proportion to the number of base pair substitutions separating the haplotypes. Color represents different countries. (H1: GGGA; H2: GAGA; H3: GGAA; H4: GGGG; H5: AGGA). Right panel shows the five haplotypes with their frequency in 473 *P. vivax* isolates.
