# Supplementary material for: Limited genetic diversity in the PvK12 Kelch protein in Plasmodium vivax isolates from Southeast Asia
Source: Malar J. 2016 Nov 8;15:537. doi: 10.1186/s12936-016-1583-0 (PMC5100195; doi:10.1186/s12936-016-1583-0)
Supplement: Supplementary file 1 — Additional file 1: Table S1. Primers used for amplification and sequencing of the full-length PvK12 gene. [file 12936_2016_1583_MOESM1_ESM.docx]

**Table S1.** Primers used for amplification and sequencing of the full-length PvK12 gene.

| **Purpose** | **Name** | **Primers** |
| --- | --- | --- |
| Primary PCR | P1F | 5’-CCATACTGGCTGCACCTGCTT-3’ |
|  | P1R | 5’-GTAGTGGCAGTGGAGGAGAG-3’ |
| Nest PCR | P2F | 5’-CCACGGAACAGATGAATCTTC-3’ |
|  | P2R | 5’-AAACCCGAGAAAGTTGTAGCA-3’ |
| Sequencing | SPF1 | 5′- CCACGGAACAGATGAATCTTC-3′ |
| Sequencing | SPR1 | 5′-CACTTGAGTCACTCAACTCCGT-3′ |
| Sequencing | SPF2 | 5′-AATGAAGATAACGCAATGAATA-3′ |
| Sequencing | SPR2 | 5′-ACTCGCTATCCCTATCTAGAA-3′ |
| Sequencing | SPF3 | 5′-GCTAAGTGGGAGGTACCACGT- 3 |
| Sequencing | SPR3 | 5′-TAGTCTTTCTCCATTGGTCCCAC-3′ |
| Sequencing | SPF4 | 5′- CGCCTCGATCTTCCTCCATGTGTG-3′ |
| Sequencing | SPR4 | 5′-AAACCCGAGAAAGTTGTAGCA-3′ |
