# Supplementary material for: Limited genetic diversity in the PvK12 Kelch protein in Plasmodium vivax isolates from Southeast Asia
Source: Malar J. 2016 Nov 8;15:537. doi: 10.1186/s12936-016-1583-0 (PMC5100195; doi:10.1186/s12936-016-1583-0)
Supplement: Supplementary file 2 — Additional file 2: Table S2. Accession numbers of Plasmodium vivax Kelch protein K12 (Pv K12) sequences retrieved from Plasmodb. [file 12936_2016_1583_MOESM2_ESM.docx]

| **Country** | **No. of samples** | **Year of sample** | **Isolate ID** |
| --- | --- | --- | --- |
| Thailand | 14 | 2012-2013 | Thailand_VKBT-36, Thailand_VKBT-37, Thailand_VKBT-39, Thailand_VKBT-45, Thailand_VKBT-52, Thailand_VKBT-71, Thailand_VKBT-72, Thailand_VKBT-94, Thailand_VKBT-95, Thailand_VKBT-98, Thailand_VKBT-99, Thailand_VKBT-100, Thailand_VKBT-101, Thailand_VKBT-106 |
| Myanmar | 5 | 2012-2013 | China_LZCH-4, China_LZCH-13,  China_LZCH-20, China_NB_16, China_NB_17 |
| Columbia | 22 | 2011-2013 | Columbia_30101099040, Columbia_30102100437, Columbia_30102100438-A, Columbia_30102100438-B, Columbia_30102100439, Columbia_30102100440, Columbia_30102100441-A, Columbia_30102100441-B, Columbia_30102100445, Columbia_30102100446, Columbia_30102100448, Columbia_30102100486, Columbia_30102100488, Columbia_30102100489, Columbia_30102100490, Columbia_30102100491, Columbia_30102100504, Columbia_30103103280, Columbia_30111110015, Columbia_30111110020, Columbia_30111110026, Columbia_30101099036 |
| Mexico | 10 | 2001-2004 | Mexico_1086-A, Mexico_161-04, Mexico_165-A, Mexico_203-04, Mexico_267-A, Mexico_32-E-03, Mexico_330-A, Mexico_566-A, Mexico_938-A, Mexico_980-A |
| Peru | 10 | 2010-2013 | Peru06, Peru07, Peru1008, Peru257, Peru260, Peru3133, DTS0791, DTS0721, DTS0839, IQ07 |
| Papua New Guinea | 3 | 1997-2012 | XUC014, PNG72, PVRVL1997 |
| North Korea | 1 | N/A | NorthKorean |
| Brazil | 1 | 2011 | Brazil |
| Mauritania | 1 | N/A | Mauritania |
| India | 2 | 2012 | IndiaNYC, IndiaVII |
| Cambodia | 284 | 2011-2013 | [35] |
| **China** | **66** | **2004-2006** |  |
| **Northeast Myanmar** | **32** | **2008** |  |
| **Western Thailand** | **22** | **2006** |  |

**Table S2.** Year of sampling and origin of all the samples used for the Haplotype network analysis and sequencing of PvK12. Samples in bold are from this study. Isolate IDs of sequences retrieved from Plasmodb are also included.
