# Supplementary material for: Limited genetic diversity in the PvK12 Kelch protein in Plasmodium vivax isolates from Southeast Asia
Source: Malar J. 2016 Nov 8;15:537. doi: 10.1186/s12936-016-1583-0 (PMC5100195; doi:10.1186/s12936-016-1583-0)
Supplement: Supplementary file 3 — Additional file 3. Alignment of the Tho2 and Cdc37 N domains. Alignment of the Tho2 and Cdc37 N domains of Plasmodium with the THo2 and Cdc37 N conserved domain family proteins. The hyphens show the gaps in the alignment. [file 12936_2016_1583_MOESM3_ESM.pdf]

```
   P.v Kelch  255 EEQKLHDERKKLDIDiSNGYKQIKKEKEEHRKRFD---EERLRFLQEIDKIKLVLYLEKEKYFQEYKN   319
 Tho2 family   32 EIEKLEKNIKELDSK-SSGIDKKKKEKKRLKSLIKklkEELKKHIEHNEKTKKRLSEEKSSWFPSKDN    98

   P.c Kelch  220 ELSDSSDFE---NMvgDLRiTFINWlKKTQMNFIREKEKLFKDKKELEME-RIRLYKEIEN-RKNIEEQ  284
CDC_37N family 11 ELSDDSDIEvhpNV--DKK-SFIRW-KQRDIHQARVERMEEIKNLKYELImNDHLNKRIDKlLKGLREE   76
   P.c Kelch  285 KLQDERKKLDIDISNGYKQIKKEKEEHRKRFDDERLRFLQEIDKIKLVLYLEKEKYFQEYKnfeNDKKKI 353
CDC_37N family 77 ELSPETPTYNEMLAELQDQLKKELEEANGDSEGLLEELKKHRDKLKKEQKELRKKLDELEK---EEKKKI 142
```
