# Supplementary material for: Limited genetic diversity in the PvK12 Kelch protein in Plasmodium vivax isolates from Southeast Asia
Source: Malar J. 2016 Nov 8;15:537. doi: 10.1186/s12936-016-1583-0 (PMC5100195; doi:10.1186/s12936-016-1583-0)
Supplement: Supplementary file 4 — Additional file 4. Multiple sequence alignment of Kelch protein from seven different Plasmodium species. Clustal Omega server was used to align the Kelch proteins from P. falciparum (PF3D7_1343700); P. reichenowi (PRCDC_1342700); P. chabaudi (PCHAS_1361300); P. berghei (PBANKA_1356700); P. yoelii (PY17X_1362400); P. knowlesi (PKNH_1257700); P. cynomolgi (PCYB_122000), P. vivax (PVX_083080). The BTB and the Kelch domains are boxed in red and blue respectively. Cysteine residues are highlighted in green. The dots indicate the break in the sequences. The BTB domains starting from 327 are shown and boxed in red. The Kelch domains are shown in blue box. The hyphens show the gaps in the alignment. [file 12936_2016_1583_MOESM4_ESM.pdf]

```
PVX_080100        ME.......IDINVGGAIFETSRHTLTQQKDSFIEKLLSGRYHVTRDKQGRIFLDRDSELFRIILNFLRNPLTVPIPKDLSESEALLKEAEFYGIK
PY17X_1241000     ME.......IDINVGGALFETSRHTLTQQKDSFIEKLLSGRYHITRDKQGRIFLDRDSELFRIILNFLRNPLTIPIPKDLGESEALLKEAEFYGIK
PBANKA_1237800    ME.......IDINVGGALFETSRHTLTQQKDSFIEKLLSGRYHITRDKQGRIFLDRDSELFRIILNFLRNPLTIPIPKDLGESEALLKEAEFYGIK
PCHAS_1238200     ME.......IDINVGGALFETSRHTLTQQKDSFIEQLLSGRYHITRDKQGRIFLDRDSELFRIILNFLRNPLTIPIPKDLGESEALLKEAEYYGIK
PF3D7_1343700     ME.......IDINVGGAIFETSRHTLTQQKDSFIEKLLSGRHHVTRDKQGRIFLDRDSELFRIILNFLRNPLTIPIPKDLSESEALLKEAEFYGIK
PRCDC_1342700     ME.......IDINVGGAIFETSRHTLTQQKDSFIEKLLSGRHHVTRDKQGRIFLDRDSELFRIILNFLRNPLTIPIPKDLSESEALLKEAEFYGIK
PKNH_1257700      ME.......IDINVGGAIFETSRHTLTQQKDSFIEKLLSGRYHVTRDKQGRIFLDRDSELFRIILNFLRNPLTVPIPKDLSESEALLKEAEFYGIK
PCYB_122000       ME.......IDINVGGAIFETSRHTLTQQKDSFIEKLLSGRYHVTRDKQGRIFLDRDSELFRIILNFLRNPLTVPIPKDLSESEALLKEAEFY---

PVX_080100        FLPFPLVFCMGGFDGVEYLNSMELLDISQQCWRMCTPMSTKKAYFGSAVLNNFLYVFGGNNYDYKALFETEVYDRLRDTWFVSSNLNIPRRNNCGV
PY17X_1241000     FLPFPLVFSIGGFDGVEYLNSMELLDISQQCWRMCTPMSTKKAYFGSAVLNNFLYVFGGNNYDYKALFETEVYDRLRDTWFLSSNLNIPRRNNCGI
PBANKA_1237800    FLPFPLVFSIGGFDGVEYLNSMELLDISQQCWRMCTPMSTKKAYFGSAVLNNFLYVFGGNNYDYKALFETEVYDRLRDTWFLSSNLNIPRRNNCGI
PCHAS_1238200     FLPFPLVFCIGGFDGVEYLNSMELLDISQQCWRMCTPMSTKKAYFGSAVLNNFLYVFGGNNYDYKALFETEVYDRLRDTWFLSSNLNIPRRNNCGI
PF3D7_1343700     FLPFPLVFCIGGFDGVEYLNSMELLDISQQCWRMCTPMSTKKAYFGSAVLNNFLYVFGGNNYDYKALFETEVYDRLRDVWYVSSNLNIPRRNNCGV
PRCDC_1342700     FLPFPLVFCIGGFDGVEYLNSMELLDISQQCWRMCTPMSTKKAYFGSAVLNNFLYVFGGNNYDYKALFETEVYDRLRDVWYVSSNLNIPRRNNCGV
PKNH_1257700      FLPFPLVFCMGGFDGVEYLNSMELLDISQQCWRMCTPMSTKKAYFGSAVLNNFLYVFGGNNYDYKALFETEVYDRLRDTWFVSSNLNIPRRNNCGV
PCYB_122000       ------VFCMGGFDGVEYLNSMELLDISQQCWRMCTPMSTKKAYFGSAVLNNFLYVFGGNNYDYKALFETEVYDRLRDTWFVSSNLNIPRRNNCGV

PVX_080100        TSNGRIYCIGGYDGSSIIPNVEAYDHRMKAWVEIAPLNTPRSSSMCVAFDNKIYVIGGTNGERLNSIEVYDEKMNKWEQFPYALLEARSSGAAFNY
PY17X_1241000     TSNGRIYCIGGYDGSSIIPNVEAYDHRMKAWIEVAPLNTPRSSAMCVAFDNKIYVVGGANGERLNSIEVYDEKMNKWENFPYALLEARSSGAAFNY
PBANKA_1237800    TSNGRIYCIGGYDGSSIIPNVEAYDHRMKAWIEVAPLNTPRSSAMCVAFDNKIYVVGGANGERLNSIEVYDEKMNKWENFPYALLEARSSGAAFNY
PCHAS_1238200     TSNGRIYCIGGYDGSSIIPNVEAYDHRMKAWIEVAPLNTPRSSAMCVAFDNKIYVIGGANGERLNSIEVYDEKMNKWEKFPYALLEARSSGAAFNY
PF3D7_1343700     TSNGRIYCIGGYDGSSIIPNVEAYDHRMKAWVEVAPLNTPRSSAMCVAFDNKIYVIGGTNGERLNSIEVYEEKMNKWEQFPYALLEARSSGAAFNY
PRCDC_1342700     TSNGRIYCIGGYDGSSIIPNVEAYDHRMKAWVEVAPLNTPRSSAMCVAFDNKIYVIGGTNGERLNSIEVYEEKMNKWEQFPYALLEARSSGAAFNY
PKNH_1257700      TSNGRIYCIGGYDGSCIIPNVEAYDHRMKAWVEIAPLNTPRSSSMCVAFENKIYVIGGTNGERLNSIEVYDEKMNKWEQFPYALLEARSSGAAFNY
PCYB_122000       TSNGRIYCIGGYDGSSIIPNVEAYDHRMKAWVEIAPLNTPRSSSMCVAFDNKIYVIGGTNGERLNSIEVYDEKMNKWEQFPYALLEARSSGAAFNY

PVX_080100        LNQIYVVGGIDNEHNILDSVEQYQPFNKRWQFLNGVPEKKMNFGAATLSDSYIITGGENGDVLNSCHFFSPDTNEWQIGPSLLVPRFGHSVLIANI
PY17X_1241000     LNQIYVVGGIDNEHNILESVEQYQPFNKRWQFLNGIPEKKMNFGATTLSDSYIITGGENGDVLNSCHFFSPDTNEWQIGPSLLVPRFGHSVLVANI
PBANKA_1237800    LNQIYVVGGIDNEHNILESVEQYQPFNKRWQFLNGIPEKKMNFGATTLSDSYIITGGENGDVLNSCHFFSPDTNEWQIGPPLLVPRFGHSVLVANI
PCHAS_1238200     LNQIYVVGGIDNEHNILESVEQYQPFNKRWQFLNGIPEKKMNFGATTLSDSYIITGGENGDVLNSCHFFSPDTNEWQIGPSLLVPRFGHSVLVANI
PF3D7_1343700     LNQIYVVGGIDNEHNILDSVEQYQPFNKRWQFLNGVPEKKMNFGAATLSDSYIITGGENGEVLNSCHFFSPDTNEWQLGPSLLVPRFGHSVLIANI
PRCDC_1342700     LNQIYVVGGIDNEHNILDSVEQYQPFNKRWQFLNGVPEKKMNFGAATLSDSYIITGGENGEVLNSCHFFSPDTNEWQLGPSLLVPRFGHSVLIANI
PKNH_1257700      LNQIYVVGGIDNEHNILDSVEQYQPFNKRWQFLNGVPEKKMNFGAATLSDSYIITGGENGDVLNSCHFFSPDTNEWQIGPSLLVPRFGHSVLIANI
PCYB_122000       LNQIYVVGGIDNEHNILDSVEQYQPFNKRWQFLNGVPEKKMNFGAATLSDSYIITGGENGDVLNSCHFFSPDTNEWQIGPSLLVPRFGHSVLIANI
```
