# Supplementary material for: Limited genetic diversity in the PvK12 Kelch protein in Plasmodium vivax isolates from Southeast Asia
Source: Malar J. 2016 Nov 8;15:537. doi: 10.1186/s12936-016-1583-0 (PMC5100195; doi:10.1186/s12936-016-1583-0)
Supplement: Supplementary file 5 — Additional file 5. Alignment of PvK12 with human KEAP1. The BTB and Kelch domains are boxed for both the proteins. The Pvk12 BTB domain is boxed in blue and Kelch domain in green; the human KEAP1 BTB domain is boxed in red and Kelch domain in pink, respectively. All the cysteines are highlighted in red. C151, C273, C288 of KEAP1 are highlighted in yellow. The hyphens show the gaps in the alignment. [file 12936_2016_1583_MOESM5_ESM.pdf]

```
PVX_080100     MEGEKIKSNSISNFSVTYERESGANSNSDDKSVSSSENESNSFMNLTSDKNEKTENNSFILNNSSFANMKDSLLESIDLSVLDSNFDSKKDFLPS    95
KEAP1_Human    MQP--------------DPRPSGA-------------GACCRFLPLQSQCPEGAGDA-----------------------VMYASTECKAEVTPS    45

PVX_080100     NLS-------------KNFNNL---------SKENLGNKYLNKLLNKSDSMFMSKGKDMNLMENNLGSNNLPVKSSNKKEGFMDSST---PINAN   165
KEAP1_Human    QHGNRTFSYTLEDHTKQAFGIMNELRLSQQLCDVTLQVKYQD----APAAQFMAHKVVL--------ASSSPVFKAMFTNGLREQGMEVVSIEGI   128

PVX_080100     EDNAMNNLKKYSNANNINDTYEKKIIETELSDSSDFENMVGDLRITFINWLKKTQMNFIREKDKLFKDKKELEMERIRLYKEIENRKSIEEQKLH   260
KEAP1_Human    HPKVMERLIEFAYTASIS-----------MGEKCVLHVMNGAVMYQ-IDSVVRACSDFLVQQL---DPSNAIG---IANFA-----EQIGCVELH   201

PVX_080100     DERKKLDIDISNGYKQIKKEKEEHRKRFDEERLRFLQEIDKIKLVLYLEKEKYF--QEYKNFENDKKKI--------VDANIATETMIDINVGGA   345
KEAP1_Human    QRAREY---IYMHFGEVAKQ----EEFFNLSHCQLVTLISRDDLNVRCESEVFHACINWVKYDCEQRRFYVQALLRAVRCHSLTPNFLQMQLQKC   289

PVX_080100     -IFETSRHTLTQQKDSFIEKLLS-----G---RYHVTRDKQGRIFLDRDSELFRIILNFLRNPLTVPIPKD-LSESEALLKEAEFYGIKFLPFPL   430
KEAP1_Human    EILQSDS-----RCKDYLVKIFEELTLHKPTQVMPCRAPKVGRLIYT-AGGYFRQSLSYLEAYN--PSDGTWLRLADLQVPRSGLAG--CVVGGL   374

PVX_080100     VFCMGGFD----GVEYLNSMELLDISQQCWRMCTPMSTKKAYFGSAVLNNFLYVFGGNNYDYKALFETEVYDRLRDTWFVSSNLNIPRRNNCGVT   521
KEAP1_Human    LYAVGGRNNSPDGNTDSSALDCYNPMTNQWSPCAPMSVPRNRIGVGVIDGHIYAVGGSHGCI-HHNSVERYEPERDEWHLVAPMLTRRIGVGVAV   468

PVX_080100     SNGRIYCIGGYDGSSIIPNVEAYDHRMKAWVEIAPLNTPRSSSMCVAFDNKIYVIGGTNGE-RLNSIEVYDEKMNKWEQFPYALLEARSSGAAFN   616
KEAP1_Human    LNRLLYAVGGFDGTNRLNSAECYYPERNEWRMITAMNTIRSGAGVCVLHNCIYAAGGYDGQDQLNSVERYDVETETWTFVAPMK-HRRSALGITV   562

PVX_080100     YLNQIYVVGGIDNEHNILDSVEQYQPFNKRWQFLNGVPEKKMNFGAATLSDSYIITGGENGDVLNSCHFFSPDTNEWQIGPSLLVPRFGHSVLIANI   712
KEAP1_Human    HQGRIYVLGGYD-GHTFLDSVECYDPDTDTWSEVTRMTSGRSGVGVAVTMEPCRKQI-----DQQNCTC---------------------------   624
```
