# Supplementary figures and images for: Limited genetic diversity in the PvK12 Kelch protein in Plasmodium vivax isolates from Southeast Asia
Source: Malar J. 2016 Nov 8;15:537. doi: 10.1186/s12936-016-1583-0 (PMC5100195; doi:10.1186/s12936-016-1583-0)

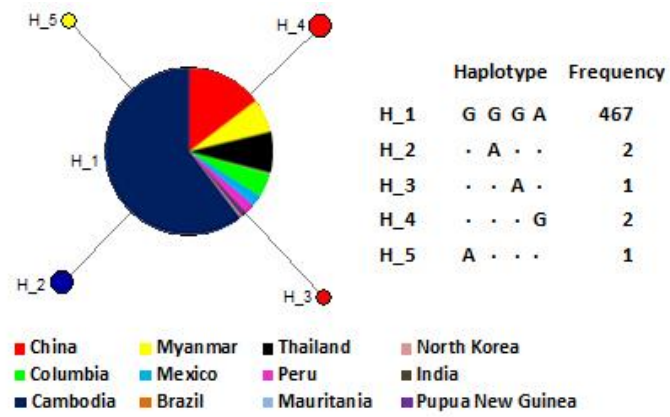

Supplement: Supplementary file 6 — Additional file 6. Haplotype network for PvK12 from global P. vivax populations. The size of the pies reflects the frequency of a particular haplotype. The lengths of the lines connecting the pies, measured from their centres, are in proportion to the number of base pair substitutions separating the haplotypes. Color represents different countries. (H1: GGGA; H2: GAGA; H3: GGAA; H4: GGGG; H5: AGGA). Right panel shows the five haplotypes with their frequency in 473 P. vivax isolates. [file 12936_2016_1583_MOESM6_ESM.pdf]
